# Vitamin D Promotes MSC Osteogenic Differentiation Stimulating Cell Adhesion and *α*V*β*3 Expression

**DOI:** 10.1155/2018/6958713

**Published:** 2018-02-28

**Authors:** Francesca Posa, Adriana Di Benedetto, Elisabetta A. Cavalcanti-Adam, Graziana Colaianni, Chiara Porro, Teresa Trotta, Giacomina Brunetti, Lorenzo Lo Muzio, Maria Grano, Giorgio Mori

**Affiliations:** ^1^Department of Clinical and Experimental Medicine, Medical School, University of Foggia, Foggia, Italy; ^2^Institute of Physical Chemistry, Department of Biophysical Chemistry, University of Heidelberg and Max Planck Institute for Intelligent Systems, Stuttgart, Germany; ^3^Department of Emergency and Organ Transplantation, University of Bari, Bari, Italy; ^4^Department of Basic and Medical Sciences, Neurosciences and Sense Organs, University of Bari, Bari, Italy

## Abstract

Vitamin D (Vit D) by means of its biological active form, 1*α*,25-dihydroxyvitamin D_3_ (1,25(OH)_2_D_3_), has a protective effect on the skeleton by acting on calcium homeostasis and bone formation. Furthermore, Vit D has a direct effect on mesenchymal stem cells (MSCs) in stimulating their osteogenic differentiation. In this work, we present for the first time the effect of 1,25(OH)_2_D_3_ on MSC adhesion. Considering that cell adhesion to the substrate is fundamental for cell commitment and differentiation, we focused on the expression of *α*_V_*β*_3_ integrin, which has a key role in the commitment of MSCs to the osteoblastic lineage. Our data indicate that Vit D increases *α*_V_*β*_3_ integrin expression inducing the formation of focal adhesions (FAs). Moreover, we assayed MSC commitment in the presence of the extracellular matrix (ECM) glycoprotein fibronectin (FN), which is able to favor cell adhesion on surfaces and also to induce osteopontin (OPN) expression: this suggests that Vit D and FN synergize in supporting cell adhesion. Taken together, our findings provide evidence that Vit D can promote osteogenic differentiation of MSCs through the modulation of *α*_V_*β*_3_ integrin expression and its subcellular organization, thus favoring binding with the matrix protein (FN).

## 1. Introduction

Vitamin D (Vit D) is well known to be important for bone health, although its mechanism of action, direct or indirect, is still a matter of debate; its effects on bone tissue and bone cells have not yet been completely clarified.

Several studies speculated on the role of Vit D in the differentiation of osteoblasts and, more recently, on mesenchymal stem cells (MSCs), which are known for their abilities in promoting bone repair and regeneration in cell reconstructive therapies [1–3].

1,25(OH)_2_D_3_, the most active form of Vit D [4], has been identified as osteoinductive, being able to promote *in vitro* the differentiation of human MSCs into osteoblasts [5, 6].

Although MSCs from bone marrow represent an ideal source of stem cells for bone regenerative therapies, their harvesting is comprehensibly complicated for patients; that is why in recent years numerous less invasive alternatives of MSCs have been proposed. Among them, multipotent stem cells from dental tissues (DSCs) have been tested as reliable candidates in tissue repair, primarily because they can be obtained from unnecessary organs such as the third molars [7–9].

Our cell model is represented by dental bud stem cells (DBSCs): postnatal MSCs isolated from the immature form of the wisdom tooth, the dental bud (DB), in children (8–12 years old). DBSCs meet all the standards to be considered MSCs, expressing more than 95% of mesenchymal stem cell markers; they can differentiate into osteoblast-like cells if cultured in an osteogenic medium (OM) [10], and this process is favored by the downregulation of the nuclear receptor NURR1 [11]; furthermore, these cells show a pattern of adhesion molecules comparable to the one described for MSCs [12]. Thus, DBSCs represent an optimal model of MSCs useful to study bone formation processes.

We have recently demonstrated that the active metabolite of Vit D, 1,25(OH)_2_D_3_, is able to stimulate the osteoblastic differentiation of DBSCs by inducing the expression of the typical osteoblastic markers and determining a higher mineralization rate *in vitro*. Moreover, the action of this molecule was particularly evident in the early stages of differentiation, decreasing over time. We concluded that Vit D acts on MSCs, driving the early phases of cell commitment toward the osteoblastic lineage [13].

Cell adhesion to the substrate is of fundamental importance for proliferation, commitment, and differentiation of MSCs [14]; no data are available at present in the literature concerning the effect of Vit D on cell adhesion molecules in MSCs, but there are evidences that the use of titanium substrates and Vit D has an additive effect in regulating the integrin expression of human osteoblast-like cells [15]; moreover, a crosstalk between the two signals, the integrin one and that induced by Vit D in promoting osteoblastic differentiation, has been hypothesized [16].

In light of this, we hypothesized that Vit D drives MSC commitment by affecting cell adhesion.

Integrins, heterodimeric transmembrane adhesion receptors, are fundamental for the extracellular matrix (ECM) assembly and, interacting with numerous extracellular and intracellular ligands, are crucial for cell fate control. As a matter of fact, as shown in different studies, the ECM contains molecules able to provide signals which on the one hand guide cell adhesion, growth, proliferation, and migration and on the other hand can define cell differentiation through the activation of integrin subunits [17–20].

Integrins, by using signaling proteins, are able to modulate both focal adhesion dynamics and cellular functions [21].

Although the specific contribution of these receptors during MSC commitment is still unclear, integrins *α*_v_*β*_3_ and *α*_5_*β*_1_ have been proven to possess key functions for bone biology: *β*_1_ integrin subfamily is predominant in osteoprogenitor cells and osteoblasts [22]; the activation of these integrins is at the basis of many processes needed for bone development on substrates, such as the formation of focal adhesions [23–25], force sensing, and mechanotransduction [26], and osteogenesis [27, 28].

*α*_v_ *β*_3_ integrin adhesion and the signals triggered by this receptor might be necessary for osteoblastic differentiation process, as proposed by Schneider et al. [20]. The expression levels of both *α*_v_ and *β*_3_ subunits, as well as their assembling to form the functional receptor, are enhanced during DBSC osteogenic differentiation [12].

It has been shown that *α*_5_*β*_1_ integrin and its interaction with fibronectin (FN), an adhesive ECM glycoprotein, are necessary for preosteoblast adhesion to the ECM and their subsequent differentiation into mature osteoblasts [29].

Hamidouche et al. [19] showed that the expression of *α*_5_ integrin is upregulated in MSCs under osteogenic conditions and that activation of this subunit is sufficient to induce osteoblastic differentiation. These observations also implicate *α*_5_*β*_1_ integrin in the control of osteoblastogenesis.

Although there are many findings supporting an involvement of these adhesion molecules in osteoblastic differentiation process, the topic is still under debate [17].

In this study, we investigated whether Vit D can influence the expression and subcellular localization of integrins in MSCs, so defining the cell fate and consequently the acquisition of osteoblastic features.

We focused on the specific expression of *α*_v_*β*_3_ integrin in DBSCs cultured on fibronectin (FN) in presence of Vit D treatment.

### 1.1. Patients, Materials, and Methods

#### 1.1.1. Materials

1*α*,25-Dihydroxyvitamin D_3_, ascorbic acid, dexamethasone, poly-L-lysine (PLL), and fibronectin (FN) were from Sigma Aldrich, St. Louis, MO, USA.

Antibody anti-*α*_V_*β*_3_ clone LM609 was from Millipore; antibodies anti-integrin *α*V and *β*3 were from BD Bioscience; anti-RUNX2 antibody was from Abnova.

The following primer pairs were used for the RT-PCR amplification: sense *Coll I* (*COL1A1*) 5′-CGTGGCAGTGATGGAAGTG-3′; antisense *Coll I* 5′-AGCAGGACCAGCGTTACC-3′; sense *RUNX2* 5′-GGAATGCCTCTGCTGTTATG-3′; antisense *RUNX2* 5′-TTCTGTCTGTGCCTTCTGG-3′; sense *OPN* (*SPP1*) 5′-CTGATGAATCTGATGAACTGGTC-3′; antisense *OPN* 5′-GTGATGTCCTCGTCTGTAGC-3′; sense *β-actin* (*ACTB*) 5′-AATCGTGCGTGACATTAAG-3′; antisense *β-actin* 5′-GAAGGAAGGCTGGAAGAG-3′; sense *β_2_ microglobulin* (*B2M*) 5′-ATGAGTATGCCTGCCGTGTGA-3′; antisense *β_2_ microglobulin* 5′-GGCATCTTCAAACCTCCATG-3′.

### 1.2. Patients and Cell Cultures

The dental buds (DBs) were collected from the third molars of 10 healthy pediatric patients aged between 8 and 12 years.

The study was approved by the Institutional Review Board of the Department of Clinical and Experimental Medicine, University of Foggia, and patients' parents gave written informed consent.

The central part of DB was cut into small pieces in a culture dish under laminar flow hood by using a sterile scalpel. Subsequently, enzymatic digestion was performed under stirring, for 1 hour at 37°C, using a 3 mg/ml solution of type I collagenase plus 4 mg/ml of dispase (Gibco Ltd., Uxbridge, UK). Single cell suspension, obtained by filtering the cells through a 70 *μ*m BD Falcon filter (Falcon) (Becton Dickinson, Sunnyvale, CA), was centrifuged at 1300 rpm for 5 min.

The resulting pellet was resuspended in a mesenchymal stem cell culture medium supplemented with 5% fetal bovine serum (FBS), 100 U/ml penicillin-G, 100 *μ*g/ml streptomycin (Gibco Limited, Uxbridge, UK). Cells seeded at a density of 5 × 10^3^ cells/cm^2^ were cultured at 37°C and 5% CO_2_, renewing the medium every 3 days.

To examine Vit D effect on cell adhesion during the osteoblastic differentiation process, 3000 cells/cm^2^ were seeded and cultured in an osteogenic medium made up of *α*-MEM supplemented with 2% FBS, 10^−8^ M dexamethasone and 50 *μ*g/ml ascorbic acid (Sigma Aldrich, St. Louis, MO, USA).

1,25(OH)_2_D_3_ (Sigma Aldrich, St. Louis, MO, USA) was reconstituted at 10^−4^ M in 95% ethanol and stored at −20°C.

The cells were grown in replicate using 1,25(OH)_2_D_3_ as treatment and an equivalent concentration of 95% ethanol as vehicle (VHC).

### 1.3. ECM Glycoproteins and Coating Procedure

Tissue culture-treated polystyrene surfaces were coated with fibronectin (FN, human plasma, 5 *μ*g/cm^2^, Sigma) diluted in 1 × phosphate buffered saline (PBS, pH 7.2, PAA, Cölbe, Germany) according to the manufacturers' suggestions. Poly-L-lysine (PLL, 2 *μ*g/cm^2^, Sigma) was used as control (CTR).

These amounts ensure the complete coating of the surface with the ECM protein (FN). The surfaces were incubated with FN solution for 30 minutes at 37°C then washed twice with PBS and blocked with bovine serum albumin (BSA, Sigma) at 1% in PBS for 10 minutes at RT. Then surfaces were sterilized in UV light for 30 minutes. The protein content of the coating solution was measured by micro-BCA assay, to ensure the correct coating adsorption and to quantify the residual protein content in the solution.

In addition, coatings were confirmed by fluorescence microscopy using labeled proteins (data not shown).

### 1.4. Immunofluorescence

A defined amount of cells was seeded and cultured on glass coverslips with the osteogenic differentiation medium, and then the cell fixation in 4% (PFA)/PBS is followed. Subsequently, the cells so treated were washed with PBS and blocked in a solution of 1% BSA and 5% normal goat serum in PBS for 20 minutes. The samples were incubated with the *α*_V_*β*_3_ antibody (clone LM609 antibody) and washed; the bound antibody was detected using 2 *μ*g/ml of fluorescently labeled goat anti-mouse secondary antibody (Alexa Fluor 488, Invitrogen); cytoskeleton was counterstained with phalloidin (Invitrogen). A multispectrum confocal microscope Leica TCS SP5 was used to visualize and photograph the cells.

### 1.5. Real-Time PCR

The extraction of total RNA was carried out utilizing spin columns (RNeasy, Qiagen, Hilden, Germany) and then in the amount of 2 *μ*g was reverse transcribed (RT) by using SuperScript First-Strand Synthesis System kit (Invitrogen Life Technologies, Carlsbad, CA, USA). An amount of 20 ng of the synthesized cDNA was subjected to quantitative PCR. Real-time PCR analysis was performed using a BioRad CFX96 Real-Time System with the SYBR Green PCR method as described by the manufacturer's protocol (BioRad iScript Reverse Transcription Supermix cat. 170-8841). The mean cycle threshold value (Ct) from triplicate samples was used to calculate gene expression, and cDNA was normalized to the average of *β*-actin and *β*_2_ microglobulin (B2M) levels for each reaction.

### 1.6. Western Blot

Revelation of *α*_V_ and *β*_3_ integrin subunits and osteoblastic markers as protein levels was performed using SDS-PAGE gel electrophoresis and Western blot analysis. Cells were lysed after 12 days of osteogenic differentiation, the lysates were centrifuged at 13000 rpm for 15 minutes at 4°C, and then a protein assay (BIORAD) was used to determine the total protein concentration of the supernatant. Proteins were separated by SDS-PAGE and then transferred to nitrocellulose membranes (Invitrogen, Carlsbad, CA). After incubation with primary and secondary antibodies, the Odyssey Infrared Imaging System of LI-COR (LI-COR Biotechnology Lincoln, Nebraska, USA) was used for immunodetection.

### 1.7. Statistical Analyses

Statistical analyses were performed by Student's *t*-test with the Statistical Package for the Social Sciences (spssx/pc) software (SPSS, Chicago, IL, USA). The results were considered statistically significant for *p* < 0.05.

## 2. Results

### 2.1. Vitamin D Treatment Induces Focal Adhesion via *α*_V_*β*_3_ Subcellular Distribution

To investigate how Vit D can influence the early stages of cell adhesion to the substrate defining, as a result, the cell fate, and consequently the acquisition of osteoblastic features, DBSCs were cultured on coated surfaces in osteogenic conditions and treated with 1,25(OH)_2_D_3_. Cells were analyzed by immunofluorescence for the subcellular distribution of *α*_V_*β*_3_ integrins.

Since DBSCs reach confluency after few days in culture-forming multilayers, the *α*_V_*β*_3_ subcellular organization was monitored after the first steps of osteogenic differentiation (3–7 days).

**Figure 1:**
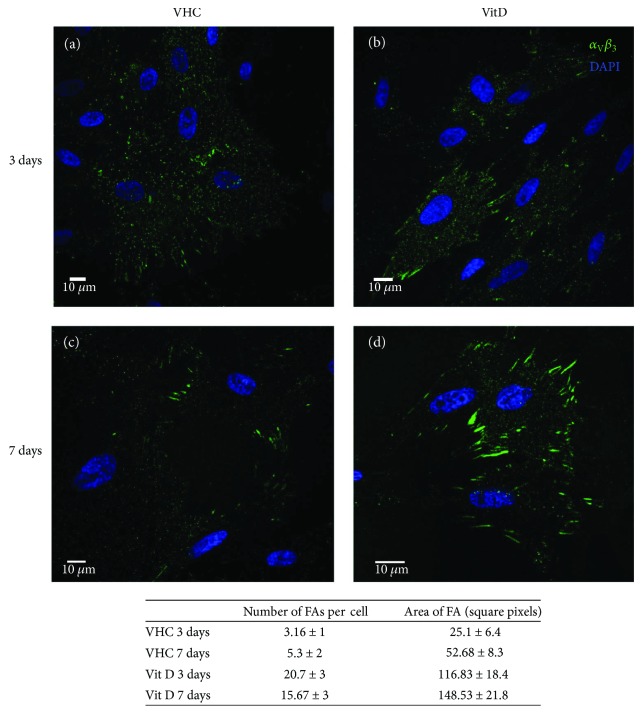
Vit D induces a clustered localization of *α*_V_*β*_3_. Midsection confocal microscopy images show the expression of integrin *α*_V_*β*_3_ (green) in DBSCs differentiated for 3 and 7 days; (a-b) show that at 3 days, the integrin was distributed in multiple sites in the cells. After 7 days of differentiation, *α*_V_*β*_3_ is localized on the periphery of the cell where the focal adhesion sites are present (c-d). Vit D treatment seemed to produce a different integrin organization leading to the formation of typical strings particularly evident after 7 days of culture. Blue for nuclei, green for *α*_V_*β*_3_. The table shows the FA quantification performed with ImageJ software. The data is presented as average ± standard error. Number of FAs per cell = total number of FAs identified in one cell. Area of FA = area of a single FA.

Vit D treatment induced a different integrin organization that appeared to be more clustered and localized in the basal part of the cell if compared to its distribution in the untreated cells (Vehicle, VHC). This effect was evident after 3 and 7 days of differentiation (Figure 1).

*α*_V_ *β*_3_ integrin was distributed throughout the cell in undifferentiated cells cultured for 3 days without Vit D; after 7 days of differentiation, it seemed to have a mild presence in focal adhesion sites (Figure 1(c)), although to a lesser extent compared to treatment with Vit D. In cells with Vit D, the integrin was clearly present and organized in FAs both at day 3 and 7 (Figures 1(b)–1(d)), with the formation of elongated clusters after one week of differentiation (Figure 1(d)).

### 2.2. Vitamin D Treatment Increases *α*_V_*β*_3_-Mediated Focal Adhesions on Fibronectin-Coated Surfaces

In order to mimic the interaction of integrins with their ECM partners, as it occurs in the bone microenvironment, we seeded DBSCs on a coating of the major cell adhesion glycoprotein: fibronectin (FN). Indeed, interaction of integrins with ECM proteins significantly induces DBSC differentiation toward osteoblastic lineage; this differentiation was enhanced when cells were grown on ECM glycoproteins containing the integrin-binding sequence, the so-called “RGD motif” [12].

To understand how Vit D treatment could affect cell adhesion in the presence of ECM glycoproteins, we prepared surfaces coated with poly-L-lysine (PLL), as control (CTR) and FN. Cells were seeded and cultured on these surfaces in osteogenic conditions for 7 or 12 days, in the presence or not of Vit D treatment, and then focal adhesion formation was analyzed looking at *α*_V_*β*_3_ subcellular distribution.

**Figure 2:**
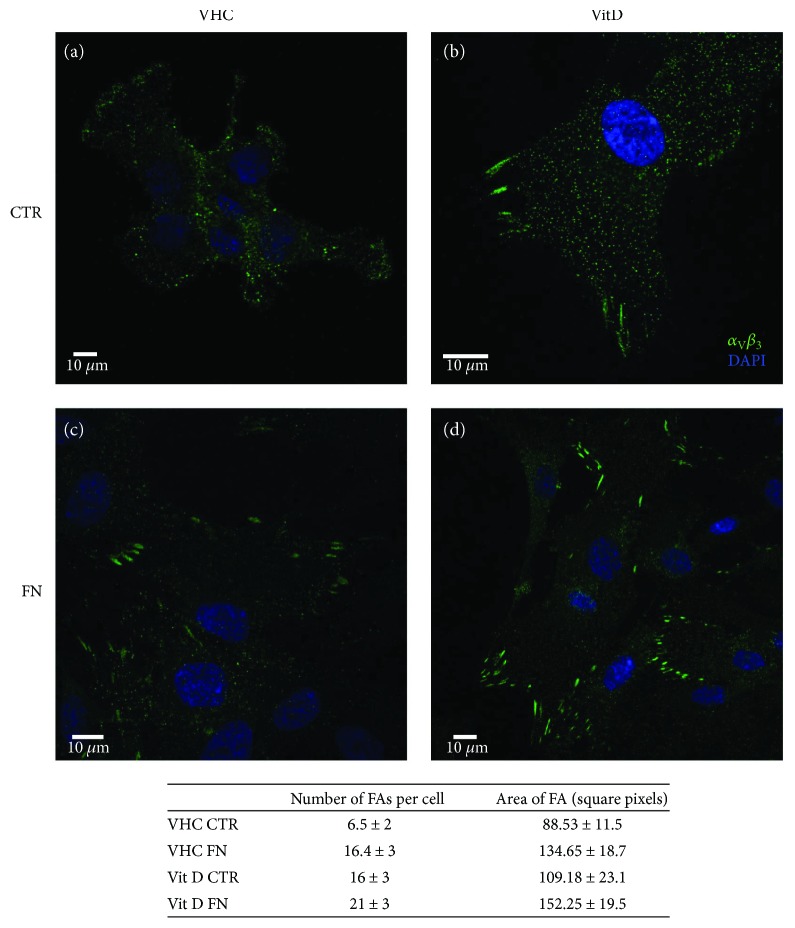
Vit D effect on *α*_V_*β*_3_ clustering is enhanced on FN-coated surfaces. Confocal images showing the expression of integrin *α*_V_*β*_3_ (green) after 7 days of osteogenic differentiation. (a-b) show the integrin distribution in cells cultured on PLL coating (CTR); in the case of untreated cells (vehicle (VHC)), *α*_V_*β*_3_ appeared to be present homogeneously in the whole cell, while Vit D treatment induced an accumulation of the receptor in the focal contacts (b–d). *α*_V_*β*_3_ clustering was increased on FN coating (c-d), more evident in Vit D treatment (d) compared to the VHC (c). Blue for nuclei and green for *α*_V_*β*_3_. The table shows the FA quantification performed with ImageJ software.

The first observation was that a higher number of cells uniformly colonized the FN-coated surface after 24 hours compared to the CTR (data not shown); furthermore, the cells seeded on FN coating showed a clustered organization of *α*_V_*β*_3_ into focal adhesions, which was sporadic in the CTR. In addition, we observed that the treatment with Vit D was able to assist the effect of the FN coating in the formation of focal adhesions. Indeed, as shown in Figure 2, the cells treated with Vit D displayed highly visible elongated clusters (typical pattern of *α*_V_*β*_3_ in FAs) if compared with untreated cells.

**Figure 3:**
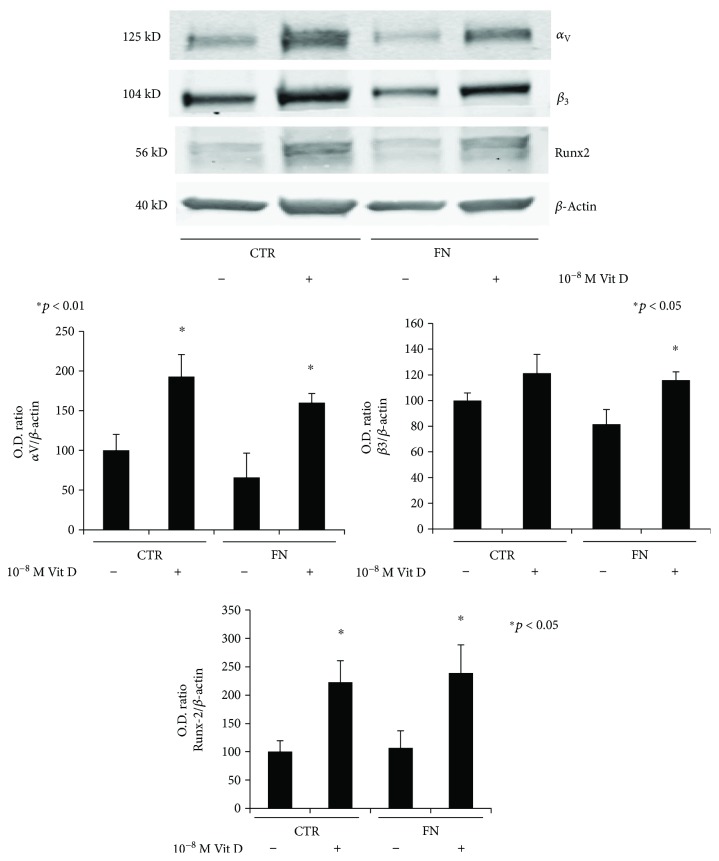
Protein expression of osteogenic markers. Immunoblots showing the expression profile of *α*_V_, *β*_3_, RUNX2, and Coll I in DBSCs cultured for 12 days in osteogenic medium with vehicle (−) and 1,25(OH)_2_D_3_ (+), on PLL-coated surfaces (CTR) or FN-coated surfaces (FN). Each graph represents means ± SEM of 3 independent experiments.

Furthermore, we evaluated the expression trend of *α*_v_ and *β*_3_ single subunits at protein level by Western blot analysis. As shown in Figure 3, Vit D treatment highly induced the protein expression of *α*_v_, which appears to be almost doubled compared to the VHC; the same is observed with respect to *β*_3_, although with a lower effect. It is clear that FN did not affect the protein expression amount while the treatment with Vit D determines a strong increase in protein expression.

### 2.3. Vitamin D Treatment Increases Osteoblast Markers Expression on Fibronectin-Coated Surfaces

In previous work [13], we demonstrated that Vit D induced the osteoblastic differentiation of DBSCs by increasing the expression of the typical osteoblastic markers. To determine if FN could influence DBSC osteoblastic features, we differentiated the cell cultures in the opportune conditions and performed a RT-PCR to evaluate the main osteoblastic markers mRNA expression.

Cells were seeded and cultured on the above-mentioned surfaces in osteogenic conditions for 12 days, in the presence or not of Vit D treatment.

**Figure 4:**
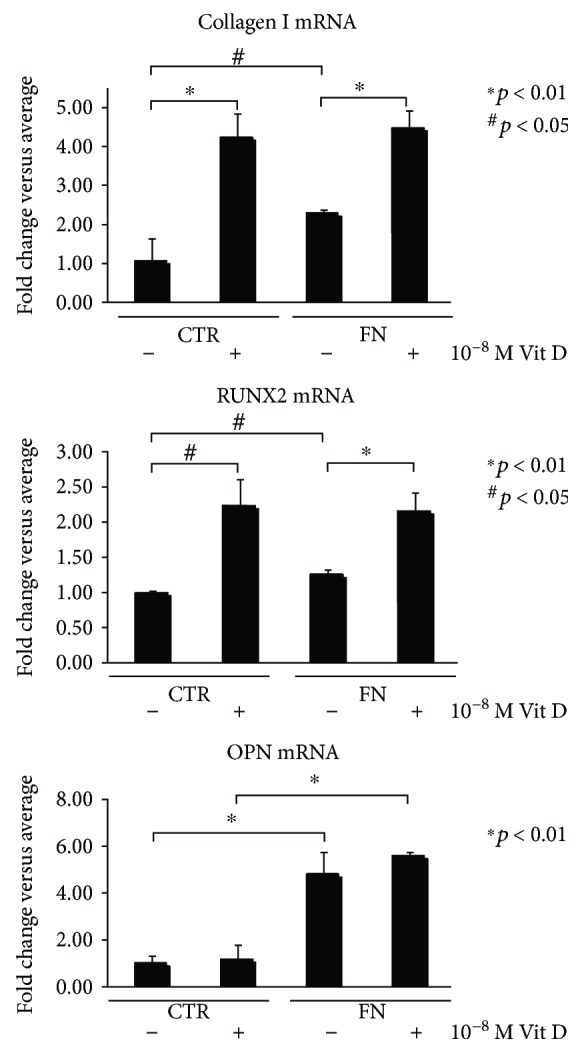
mRNA expression of osteogenic markers. qPCR analysis of Coll I, RUNX2, and OPN performed on DBSCs after 12 days of osteogenic differentiation using a medium with vehicle (−) and 1,25(OH)_2_D_3_ (+), on PLL-coated surfaces (CTR) or FN-coated surfaces (FN). Expression was normalized to the average of *β*-actin and *β*_2_ microglobulin (B2M) levels for each reaction. ^∗^*p* < 0.01 and ^#^*p* < 0.05.

Figure 4 shows that Coll I and RUNX2 mRNA levels greatly increased in the cells treated with Vit D, on control surfaces as well as on FN coating, corroborating that Vit D is able to determine the acquisition of the typical osteoblastic features in DBSCs cultured in osteogenic medium.

The effect of FN emerged by looking at the untreated samples; indeed, a significant induction of Coll I and RUNX2 mRNA expression was also observed on FN coating, when compared to CTR.

Obviously, due to the prominent effect of Vit D, the inductive influence of FN, emerged in the untreated samples, was quenched when the cells were treated with Vit D, resulting in no significant differences.

A similar trend to the one described for RUNX2 mRNA was observed at the protein level. Western blot analysis, performed after 12 days of culture, showed an increase in RUNX2 expression due to the action of Vit D, but no appreciable variation in response to FN substrate (Figure 3).

Contrary to what was observed for Coll I and RUNX2, the increase in protein expression levels of osteopontin (OPN) can be attributed to the coating with FN and only to a lesser extent to the Vit D treatment. OPN expression in untreated cells on FN showed a 4-fold increase relative to CTR and upregulation, even more in presence of Vit D, showing more than 5-fold increase compared to CTR (Figure 3). These results are in line with those obtained in our previous report [13], according to which no particular effect on OPN expression may be attributed to the vitamin.

## 3. Discussion

There are not many data in the literature about the influence of Vit D in cell adhesion, but it is well known that interactions between cells and surfaces are involved in the activation of a series of signals that in turn are responsible for cell commitment and differentiation. Thus, a recent *in vivo* study indicated that Vit D administration decreased the serum levels of the intracellular adhesion molecules L-CAM-1 and V-CAM-1 in hemodialysis patients [30]. Since the few data available are referred only to osteoblast-like cells [15, 16], we tried to determine if Vit D might, or not, have a role also in cell adhesion mechanisms of MSCs.

In this work, we analyzed the effect of Vit D on *α*_V_*β*_3_ integrin expression and subcellular organization in DBSCs during their osteogenic differentiation. Moreover, the effect of the molecule was evaluated on cells growing on the ECM glycoprotein FN.

We first cultured DBSCs on normal surfaces, under differentiating conditions, in the presence or not of Vit D, with the purpose to investigate whether this factor could have an effect on this integrin that has been already demonstrated to be involved in the osteogenic commitment of MSCs [12].

In this previous work, we determined that the interaction of DBSCs with ECM glycoproteins increased the osteogenic commitment and we showed that *α*_V_*β*_3_ integrin assumed a key role in this result; in fact, the perturbation of this receptor led to a reduction of both the alkaline phosphatase (ALP) expression and the mineralization process. In the light of this knowledge, we focused our attention on *α*_V_*β*_3_ distribution in DBSCs cultured in the presence of Vit D.

Our data indicated that *α*_v_*β*_3_ was expressed in DBSCs cultured under osteogenic conditions, and its localization underwent changes with the advancement of the osteogenic differentiation in untreated cells, but the Vit D treatment, interestingly, enhanced *α*_v_*β*_3_ accumulation in clusters corresponding to the adhesion sites represented by FAs (Figure 1).

Although there are many different types of integrins with specificity to different ECM proteins, a large number of cellular and biophysical studies have focused on *α*_5_*β*_1_ and *α*_v_*β*_3_ integrins and some of them identified in *α*_v_*β*_3_ a valid substitute to *α*_5_*β*_1_ for fibronectin binding [31–33]. We cultured our cell model of MSCs on FN-coated surfaces and studied the effects of Vit D on these integrin-glycoprotein interactions during osteogenic differentiation. DBSCs created a uniform monolayer after few days of culture, but we observed that the cells seeded on FN exhibited a more flattened morphology and seemed to be more numerous compared to the CTR (data not shown).

Integrins bind the ECM through their extracellular domains. Subsequently, their cluster and their short cytoplasmic tails interact with intracellular molecules for signal transduction pathway [34], giving rise to focal adhesions (FAs) [35].

Stable FAs were highlighted by immunofluorescence with *α*_v_*β*_3_ antibody. After 7 days in culture, DBSCs treated with Vit D on FN-coated surfaces showed large and discrete *α*_v_*β*_3_-positive clusters while a lower number of *α*_v_*β*_3_-containing complexes could be seen in untreated cells (Figure 2).

Our findings revealed a high adhesive interaction between MSCs and FN, as also observed in other researches, confirming the mesenchymal features of DBSCs [25, 36–39].

Thus, these results are consistent with a recent study in which it has been proven that FN coating can be considered able to induce *α*_v_*β*_3_ integrin expression in MSCs [40]. Our study goes forward identifying in Vit D a further support to the effect of FN by increasing FA formation. Our data confirmed that FN is capable of organizing *α*_v_*β*_3_ integrin in FAs and, above all, they indicated that Vit D leads to a significant enhancement in the receptor subunit expression, contributing to its organization in clearly visible strips.

This detail is highly relevant because it is known that effective adhesion is strictly connected with cell differentiation: in particular, osteogenesis needs a large number of FAs, while both adipogenesis and chondrogenesis are promoted when the formation of strong FAs is prevented [41].

It has been previously demonstrated that DBSC osteogenic differentiation is increased by Vit D, [13]: we hypothesized that the effect of Vit D in inducing osteoblastic differentiation in our cell model could have been mediated by an effect of the vitamin on cell adhesion. Thus, we investigated FA aspect during DBSC osteoblastic differentiation in cultures incubated with Vit D: in particular, we studied its ability to act on the expression of *α*_v_*β*_3_ integrin which plays a pivotal role during the commitment of MSCs to osteoblast lineage. Vit D prompts the expression of *α*_v_*β*_3_ integrin in turn favoring the formation of FAs, peculiar for MSC commitment and osteogenic differentiation: this finding is supported by the increased expression of RUNX2 and collagen I, two of the main early osteoblastic markers.

In conclusion, our results identified *α*_v_*β*_3_ integrin as the possible mediator of Vit D effect on MSC commitment into osteoblast-like cells further demonstrating that Vit D enhanced the interaction of *α*_v_*β*_3_ with its ECM partner FN.

The nature of FN action towards *α*_v_*β*_3_ in MSCs is just to promote cell adhesion; in fact, FN induces integrin clustering but has no effect on its expression.

Indeed, Vit D is responsible for the *α*_v_*β*_3_ integrin protein expression, and this is the point of force of our work: Vit D determines MSC commitment to the osteogenic lineage precisely through a modulation of the integrin receptor expression, resulting in the binding to its corresponding matrix protein.
