# Erratum to “Vitamin D Promotes MSC Osteogenic Differentiation Stimulating Cell Adhesion and *α*V*β*3 Expression”

**DOI:** 10.1155/2018/1865084

**Published:** 2018-09-27

**Authors:** Francesca Posa, Adriana Di Benedetto, Elisabetta A. Cavalcanti-Adam, Graziana Colaianni, Chiara Porro, Teresa Trotta, Giacomina Brunetti, Lorenzo Lo Muzio, Maria Grano, Giorgio Mori

**Affiliations:** ^1^Department of Clinical and Experimental Medicine, Medical School, University of Foggia, Foggia, Italy; ^2^Institute of Physical Chemistry, Department of Biophysical Chemistry, University of Heidelberg and Max Planck Institute for Intelligent Systems, Stuttgart, Germany; ^3^Department of Emergency and Organ Transplantation, University of Bari, Bari, Italy; ^4^Department of Basic and Medical Sciences, Neurosciences and Sense Organs, University of Bari, Bari, Italy

In the article titled “Vitamin D Promotes MSC Osteogenic Differentiation Stimulating Cell Adhesion and *α*V*β*3 Expression” [1], the text reading “The publication of this article was funded by Max Planck” was included by mistake and has been removed from the article. Additionally, the following sentence was missing from the Acknowledgments section and has been added in place:

The Article has been published with the contribution of 5x1000 IRPEF funds in favor of the University of Foggia, in the memory of Gianluca Montel.
